# Re-description of 1^st^ instar larva of *Osmylusfulvicephalus* (Scopoli, 1763) (Neuroptera, Osmylidae) based on naming system of sclerites

**DOI:** 10.3897/BDJ.12.e129392

**Published:** 2024-09-13

**Authors:** Haohan Mao, Zhiqi Liu, San-An Wu, Han Xu

**Affiliations:** 1 Beijing Forestry University, Beijing, China Beijing Forestry University Beijing China; 2 China Agricultural University, Beijing, China China Agricultural University Beijing China

**Keywords:** lance lacewing, larva, morphology

## Abstract

**Background:**

*Osmylusfulvicephalus* (Scopoli, 1763), the type species of the Osmylidae, has garnered significant research interest in taxonomy, particularly regarding its larval stages. To date, most studies have focused on the third instar larvae, but ignore the first instar larvae with the rough and incomplete morphological descriptions. This lack of studies has led to an unclear understanding of the morphological differences between larval stages, making it challenging to identify larvae, distinguish different osmylid species or even reconstruct phylogeny.

**New information:**

In this study, the first instar larva of *O.fulvicephalus* was re-described in detail and illustrated, based on the naming system of sclerites. Based on this, it could be effectively distinguished from other species by the following characteristics: i) number of segments in the labial palpi; ii) the setae beard on the apex of antenna; iii) the number of transversal rows of the anal hooks. In addition, we compared the sclerites and other morphological characteristics with the third instar larvae of *Osmylusfulvicephalus* to better distinguish larvae at different developmental stages.

## Introduction

The type species of the family Osmylidae, *Osmylusfulvicephalus* Scopoli, 1763, is widely distributed in Europe (Cover and Resh 2008). The adult stage of this species was studied in terms of its taxonomy and ecology (Scopoli 1763, Meinander 1962, Aspöck et al. 1980, Monserrat 2014). Meanwhile, its larval stage, which plays a crucial role in its life cycle, had also garnered considerable attention since its first description by Brauer (1851).

The predatory nature of the larvae of *O.fulvicephalus* is well-documented, with reports of them preying on tipulid and chironomid larvae (Brauer 1851, Withycombe 1922, Ward 1965, Gepp 1984, Monserrat 2014, Cover and Bogan 2015). Due to frequent sightings at the edge of streams or near moist zones, some researchers have suggested an aquatic habit for the larvae (Brauer 1851, Girard 1879, Withycombe 1922). However, other scholars maintain that they are terrestrial (Brocher 1913, Lestage 1920, Ward 1965). As holometabolous insects, the duration of each larval instar of *O.fulvicephalus* is influenced by environmental temperature and prey availability, with the second or third instar larvae typically overwintering and pupating in April to May of the following year (Withycombe 1922, Gepp 2003).

In terms of taxonomy, the larvae of this species are thoroughly studied, with lots of delicate illustrations and are better known than other osmylid species (Brauer 1851, Hagen 1852, Withycombe 1922, Rabaud 1927, Killington 1936, Wundt 1961, Gepp 2003, Cover and Bogan 2015). However, these studies mainly focused on the third instar larva and largely ignored the first instar. Hagen (1852) noted the large sclerite present on each side of the dorsum of meso- and metathorax of the first larva and five rows of hooks on the anal apparatus. Lestage (1920) provided accurate illustrations of both first and third instar larvae, but he did not describe them. Then, Withycombe (1922), Withycombe (1924) only briefly described the first instar larva and suggested that the morphological differences between the first instar and the third one were the fewer setae and the shape of the empodium. Gepp (2003) provided lots of drawings and ecological photos of larvae, but little information about the morphology of the first instar larva. In brief, the description of the first instar larva of *O.fulvicephalus* has been rough and incomplete, especially with characters of the ventral side abdomen often overlooked. Although these studies have advanced our understanding of the first instar larva, they have provided too little morphological information to identify larvae, distinguish different osmylid species or even reconstruct phylogeny.

In this study, we re-described the 1^st^ instar larva of *Osmylusfulvicephalus* in detail, based on the sclerites naming system (Matsuno and Yoshitomi 2016).

## Materials and methods

Two larval specimens studied here were hatched from eggs laid by a female adult of *Osmylusfulvicephalus* (Scopoli, 1763) collected from surroundings of Frauenberg Hostel, Freyung, Bayern in Germany (48,79423°N 13.7584°E) on 26 June 2018.

The larval specimens for observation structures were cleaned out by injecting 10% potassium hydroxide (KOH) solution and moved to pure glycerine. The observation, description and drawing are based on the fully expanded specimens in glycerine. The nomenclature of the 1^st^ instar larva refers to Matsuno and Yoshitomi (2016).

## Taxon treatments

### Osmylus fulvicephalus

(Scopoli, 1763)

F07B336E-8AFA-58FD-8C50-67B2529131E4

#### Materials

- **Type status:** Other material. **Occurrence:** recordedBy: Xu Han; individualCount: 2; sex: exs; lifeStage: first instar larva; occurrenceID: B616DC45-2212-5973-878D-7264A0E78B72; **Taxon:** scientificName: Osmylusfulvicephalus; **Location:** country: Germany; stateProvince: Bayern; locality: surroundings of Frauenberg Hostel, Freyung, Bayern in Germany; decimalLatitude: 48.7942; decimalLongitude: 13.7584; georeferenceProtocol: GPS; **Record Level:** collectionCode: Insects; basisOfRecord: PreservedSpecimen

#### Description

**Figure 1.**
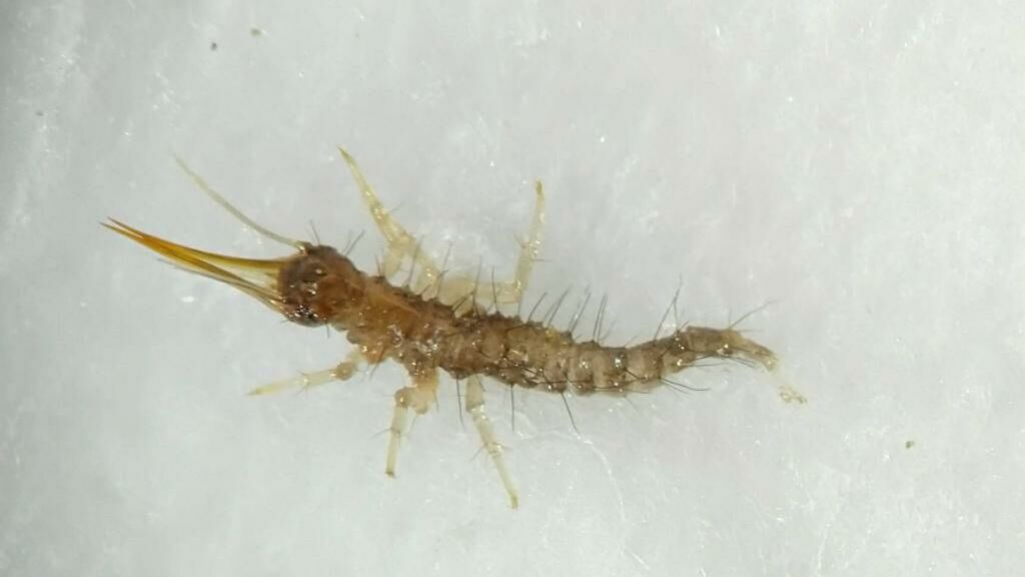
The first instar larva of *Osmylusfulvicephalus* photographed in the laboratory rearing environment.

Colouration. Head capsule brown, except for black coloured ocular region; legs light yellow; dorsal side of thorax and abdomen basically light yellow to brown (Fig. 1).

**Figure 2.**
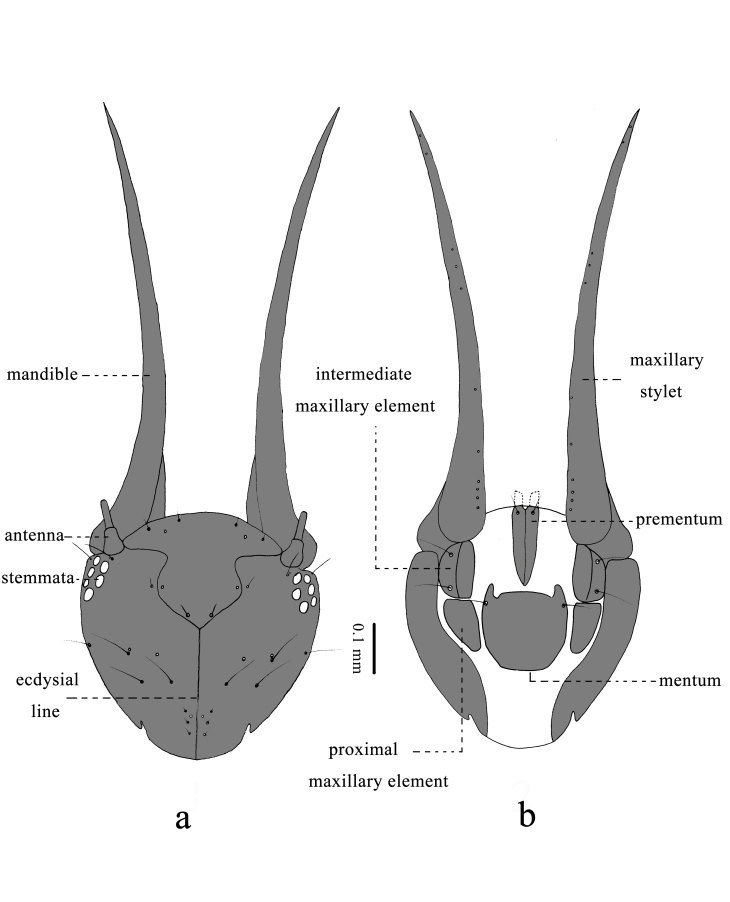
Head capsule of *Osmylusfulvicephalus*. **a** Dorsal view; **b** Ventral view.

Head capsule (Figs 2, 3): Cranium bulb-like shaped, strongly sclerotised dorsally and laterally, surface smooth. Anterior edge of labrum with two pairs of short setae and two pores; frontal edge of ocular region with two long setae; posterior margin of acetabulum of antenna with two long setae and two pores; antenna (Fig. 3a) four-segmented, first segment trapezoidal, with two pores, second segment annulated, much longer than first one, third segment little longer than first one, with a pore and a long seta on median area, terminal segment short, apex narrow, bearing a long and two short setae; frons with two pairs of short setae and two pores along ecdysial line; four pairs of long setae and two pairs of pores on vertex; three pairs of short setae and a pair of pores on occiput. Mandible long, curved outwards and upwards, without distinct teeth, except for minute serrations at apex. Maxillary stylet long and smooth, without any serration and palpi, swollen at basal portion, bearing 11 pairs of pores. Intermediate maxillary elements ellipsoidal, attached to posterior margin of maxillary stylets, each laterally with two long setae. Proximal maxillary element similar to intermediate maxillary one in shape, without seta. Mentum large, antero-lateral angles protruding, with two long setae. Prementum inverted triangular, with two long setae. Labial palpi (Fig. 3b) four-segmented, first segment long distally with a long seta, a short seta and a pore; second one short without any seta and pore; third one short, slightly longer than second one, distally with two short setae; fourth segment long, gently tapered apically.

**Figure 4.**
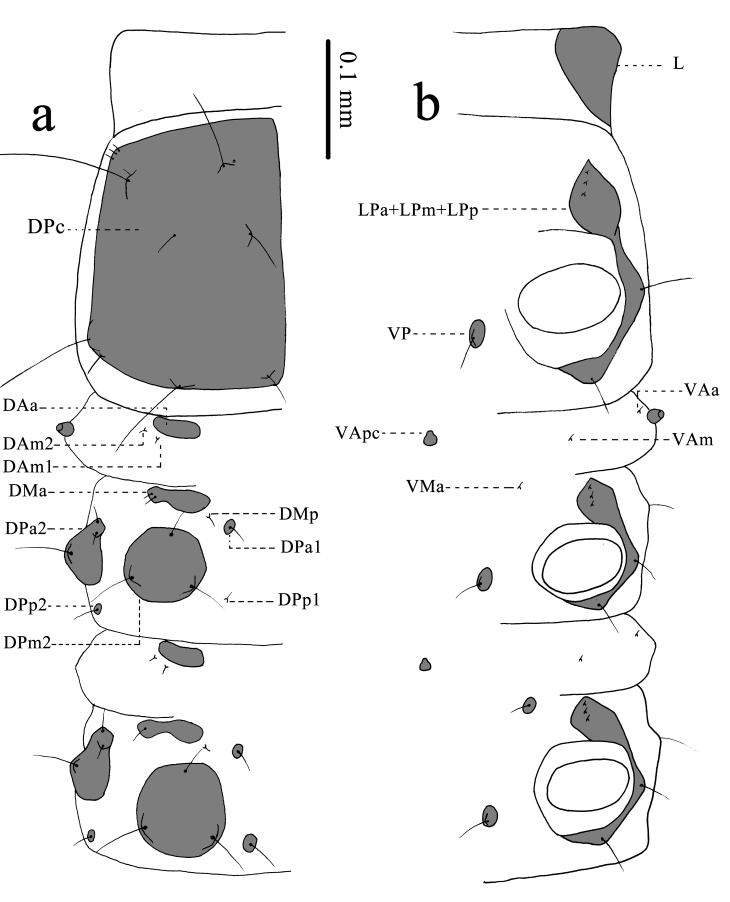
Thorax of *Osmylusfulvicephalus*. **a** Dorsal view; **b** Ventral view.

**Thorax** (Fig. 4) wider than head capsule, with numerous sclerites. Cervix (Fig. 4) narrower than cranium, very elastic, provided with a set of sclerites (paired L). L large, located in ventrolateral part, without any seta and pore.

**Prothorax** (Fig. 4): provided with three sets of sclerites (DPc; paired LPa+LPm+LPp, paired VP). DPc very large, located in the prothorax dorsally, with three pairs of short setae and two pairs of long setae anteriorly; a pair of long setae and a pair of middle setae medially; two pairs of middle setae and two pairs of long setae posteriorly. LPa+LPm+LPp large and long, three-branched, its anterior upside branch bearing three very short setae, middle branch attached with coxal cavity with a long seta and posterior branch bearing a long seta, located dorsally in coxal cavity. VP small, located in the interior part of coxal cavity, bearing a long seta.

**Mesothorax** (Fig. 4): provided with 16 sets of sclerites (paired DAa, paired DAm1, paired DAm2, paired DMa, paired DMp, paired DPa1, paired DPa2, paired DPm2, paired DPp1, paired DPp2, paired LPa+LPm+LPp, paired VAa, VApc, paired VAm, paired VMa, paired VP). DAa large, located in the anterior margin of mesothorax dorsally, without any seta. DAm1 and DAm2 very small, located in the exterior and posterior part of DAa, each bearing a short seta. DMa large, located in the posterior part of DAm1, with two short setae. DMp very small, located in the interior and posterior part of DMa, bearing a short seta. DPa1 small, located in the interior and posterior part of DMp, bearing a long seta. DPa2 large, located in the exterior and posterior part of DMa, bearing a long seta and two short setae. DPm2 large, located in the posterior part of DMa, bearing three long setae. DPp1 small, located in the posterior part of DPa1, bearing a short seta. DPp2 small, located in the posterior part of DPa2, bearing a long seta. LPa+LPm+LPp large and long, three-branched, its anterior upside branch bearing three very short setae, middle branch attached with coxal cavity with a long seta and posterior branch bearing a long seta, located dorsally in coxal cavity. VAa small, located in lower part of spiracle, bearing a short seta. VAm small, located in the interior part of VAa, bearing a short seta. VApc small, located in the anterior margin of mesothorax centrally, without any seta. VMa very small, located in the exterior and posterior part of VApc, bearing a short seta. VP small, located in the interior part of coxal cavity, bearing a long seta.

**Metathorax** (Fig. 4) similar to mesothorax in conformation of sclerites, provided with 16 sets of sclerites (paired DAa, paired DAm1, paired DAm2, paired DMa, paired DMp, paired DPa1, paired DPa2, paired DPm2, paired DPp1, paired DPp2, paired LPa+LPm+LPp, paired VAa, VApc, paired VAm, paired VMa, paired VP). DMa bearing only a short seta. DPp1 large, bearing a long seta.

**Figure 5.**
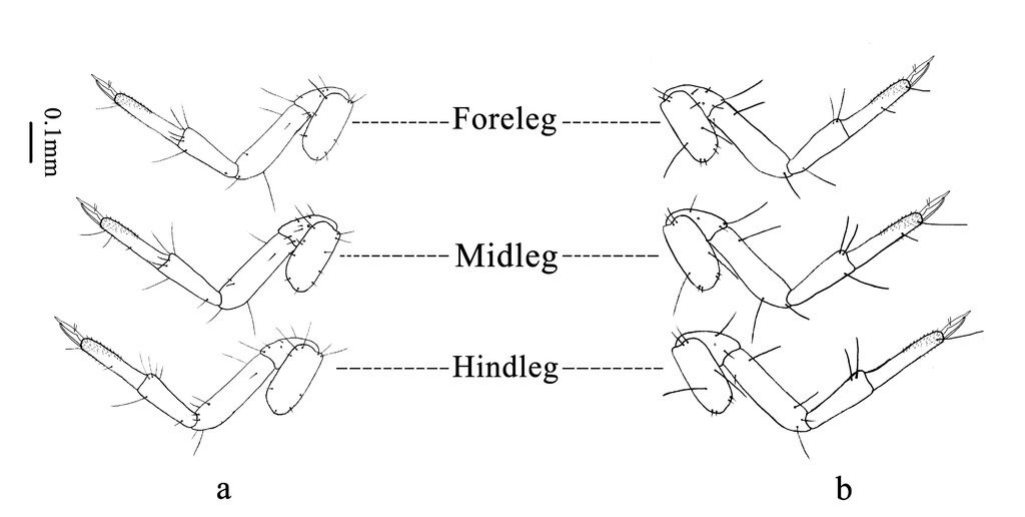
Legs of *Osmylusfulvicephalus*. **a** Interior surface of legs; **b** Exterior surface of legs.

**Legs** (Fig. 5) well developed. Fore-leg (Fig. 5): coxa quadrate, bearing four long setae and four short setae on the exterior face, three long setae and five short setae on the interior face. Trochanter subtriangular, bearing a long seta, four short setae and a pore on the exterior face, a long seta, three short setae and two pores on the interior face. Femur long, exterior face with a long seta and a short seta on the proximal half and a long seta on the distal half, interior face with a short seta on the proximal half and a long seta and a short seta on the distal half. Tibia long, exterior face with a long seta on the proximal half and two long setae on the distal half, interior face with a pore on the proximal half and three long setae on the distal half. Tarsomeres fused forming one cylindrical segment, exterior face with a short seta, a long seta and many very short hairs at apex; two tarsal claws and one empodium long and triangular, bearing two short setae. Mid-leg (Fig. 5): coxa quadrate, bearing four long setae and four short setae on the exterior face, three long setae, seven short setae and a pore on the interior face. Trochanter subtriangular, bearing two long setae, a short seta and a pore on the exterior face, six short setae and a pore on the interior face. Femur long, exterior face with a long seta on the proximal half and two long setae on the distal half, interior face with a short seta on the proximal half and two short setae on the distal half. Tibia long, exterior face with three long setae on the distal half, interior face with a short seta on the proximal half and two long setae on the distal half. Tarsomeres fused forming one cylindrical segment, bearing many very short hairs at apex, exterior face with a short seta, a long seta and interior face with a short seta in the middle; two tarsal claws and one empodium long and triangular, bearing two short setae. Hind-leg (Fig. 5): coxa quadrate, bearing four long setae and four short setae on the exterior face, four long setae and five short setae on the interior face. Trochanter subtriangular, bearing a long seta, a short seta and a pore on the exterior face, a long seta, two short setae and a pore on the interior face. Femur long, exterior face with a long seta and a short seta on the proximal half and two long setae and a short seta on the distal half, interior face with a short seta on the proximal half and a long seta and two short setae on the distal half. Tibia long, exterior face with two long setae on the distal half, interior face with a short seta on the proximal half and three long setae on the distal half. Tarsomeres fused forming one cylindrical segment, bearing many very short hairs at apex, exterior face with a short seta and a long seta and interior face with a short seta in the middle; two tarsal claws and one empodium long and triangular, bearing two short setae.

**Figure 6.**
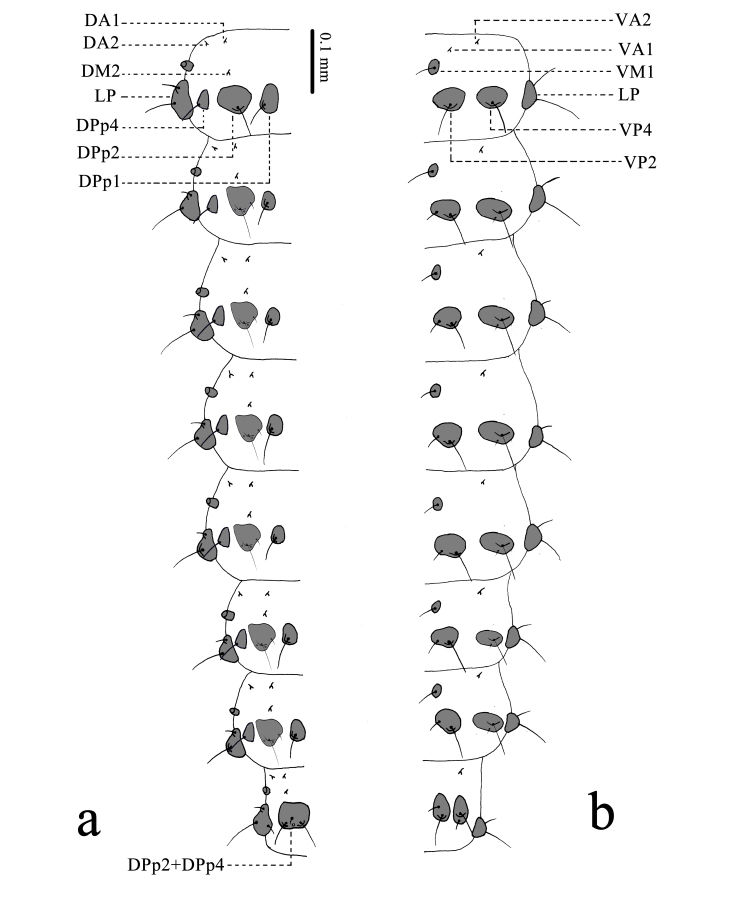
First to eighth abdominal segments of *Osmylusfulvicephalus*. **a** Dorsal view; **b** Ventral view.

**Figure 7.**
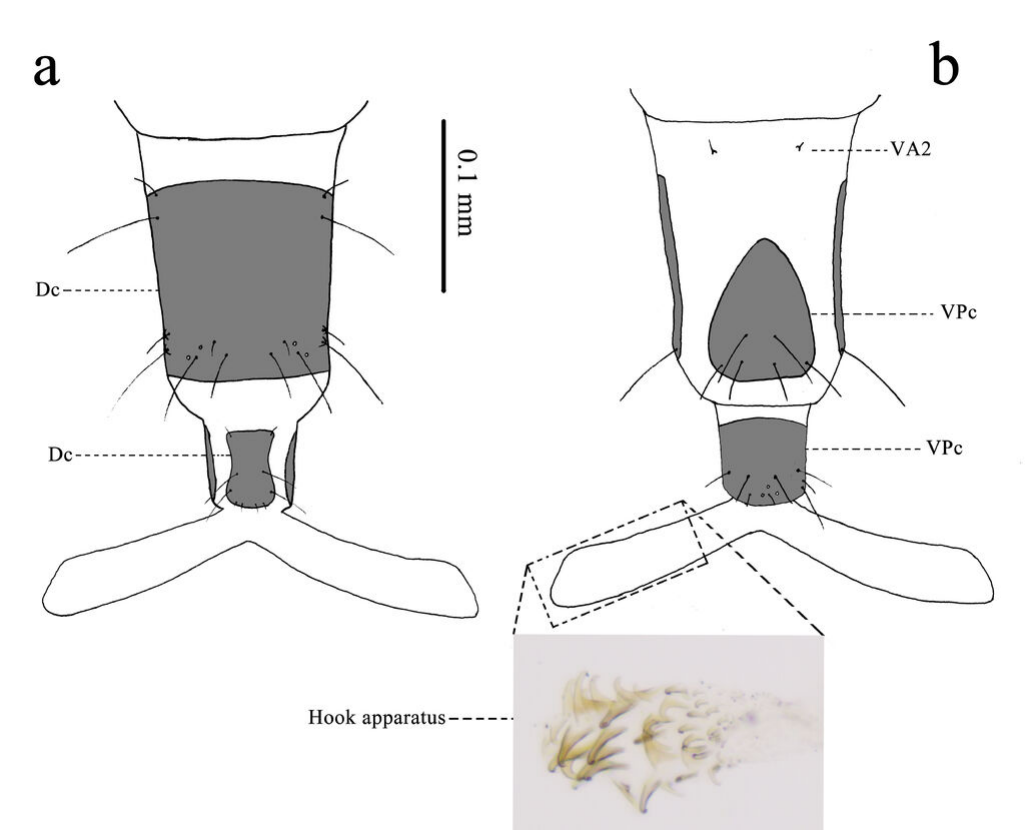
9^th^ to 10^th^ abdominal segments of *Osmylusfulvicephalus*. **a** Dorsal view; **b** Ventral view.

**Abdomen** (Figs 6, 7) 10-segmented; spiracles present from first to eighth segments, each one partly sclerotised dorsolaterally. First abdominal segment: provided with 12 sets of sclerites (paired DA1, paired DA2, paired DM2, paired DPp1, paired DPp2, paired DPp4, paired LP, paired VA1, paired VA2, paired VM1, paired VP2, paired VP4). DA1 and DA2 very small, located in the anterior part of segment dorsally, each bearing a short seta. DM2 small, located in the posterior part of DA1, bearing a short seta. DPp1 large, located in the intero-posterior part of DPp2, bearing a long seta. DPp2 large, located in the posterior part of DM2, bearing a long seta. DPp4 large, size similar to DPp1, located in the exterior part of DPp2, bearing a long seta. LP large, located in the posterolateral part, bearing a long seta and a medium seta. VA1 and VA2 very small, located in the anterior part of segment ventrally, each bearing a short seta. VM1 approximately oval, located in the posterior and interior part of VA1, bearing a long seta. VP2 and VP4 large, located in the posterior part of VM1, each bearing a long seta. Second to seventh abdominal segment: similar to first one in arrangements of sclerites, provided with 11 sets of sclerites (paired DA1, paired DA2, paired DM2, paired DPp1, paired DPp2, paired DPp4, paired LP, paired VA2, paired VM1, paired VP2, paired VP4). DPp2 bearing a long seta, a medium seta and two pores. VP2 and VP4 each bearing a long seta and a medium seta. Eighth abdominal segment: similar to the seventh in conformation of sclerites, provided with eight sets of sclerites (paired DA1, paired DA2, paired DM2, paired DPp1+DPp2, paired LP, paired VA2, paired VP2, paired VP4). DPp1+DPp2 large, bearing two long setae, a medium seta and a pore. DPp4 absent. LP bearing a long seta and two medium setae. VM1 absent. Ninth abdominal segment: provided with three sets of sclerites (Dc, VPc; paired VA2). Dc large, widely covering dorsalaterally, bearing two long setae and two short setae anteriorly; four short, six long setae and four pores posteriorly. VA2 small, located in the antero-ventral part, bearing a short seta. VPc large, triangular, bearing six long setae. 10^th^ abdominal segment: provided with two sets of sclerites (Dc, VPc). Dc large, located in the segment ventral-posteriorly, bearing two short setae anteriorly; two long setae in the middle; two long setae and four short setae posteriorly. VPc large, widely covering ventrally, bearing five long setae, three short setae and three pores. Hook apparatus (Fig. 7) bearing nine lines of hooks on ventral surface of posterior half; hooks gradually becoming smaller basally.

#### Remarks

The morphology of the first instar larva of *O.fulvicephalus* differs greatly from the first instar larva of *Stenosmylustenuis* (Walker, 1853) which was described by New (1974). The labial palpi of first instar larva of *O.fulvicephalus* is four-segmented, while three-segmented in latter species. The terminal segment of the antenna of *O.fulvicephalus* has a long and apical seta and two short setae while a long and apical seta and six short setae are present on that of latter species. Besides, the anal hooks of first instar larvae of *O.fulvicephalus* are arranged in nine transversal rows, while there is only a transversal row on anal apparatus of *S.tenuis*.

Based on the description of the third instar larva of *Osmylusfulvicephalus* recorded by previous studies (Brauer 1851, Hagen 1852, Withycombe 1922, Rabaud 1927, Gepp 2003, Monserrat 2014) and our unpublished data, the morphological differences between the first and third instar larva of *O.fulvicephalus* are more than the numbers of setae and shape of empodium and the differences in the following morphological characteristics.

On the head capsule, two pairs of short setae are located along the ecdysial line of the first instar larva, while three pairs are present along that of the third instar larva. In addition, there are only two long setae present around the ocular region on each side, but three long setae present on the corresponding position of the third instar larva. The labial palpi of the 1^st^ instar larva is four-segmented, but five-segmented in the 3^rd^ instar larva.

On the thorax, the sclerite L on cervix of the first instar larva has no seta while a long seta presents this sclerite of the third instar larva. Three sets of sclerite DA1, DA2 and DM on the prothorax of the first instar larva are fused with DPc, but these sclerites are separated on that of the third instar larva. Moreover, the sclerites VAa and VAp on the prothorax of the first instar larva are absent while present on that of the third instar larva. The sclerite DPp1 on mesothorax of the first instar is much smaller than DPa1 while that of the third instar is approximately equal to Dpa1 in size. VMp1 and VMp2 (mesothorax and metathorax) absent (existed). The sclerites LAa, LAm and LAp on the mesothorax and metathorax of 1^st^ instar larva are fused, but separated in the 3^rd^ instar larva. These three sets of sclerites are also separated in other osmylid larvae (Matsuno and Yoshitomi 2016, Martins et al. 2018). On the legs, the empodium of the first instar larva is basally equipped with two slender setae, but that of the third instar is distally with numerous short setae as illustrated by Hagen (1852).

The dorsal side of abdomen is characterised by sclerite DM2 on each of 1^st^ to 8^th^ abdominal segments of 1^st^ instar larva, much smaller than DPp1, but same as DPp1 of 3^rd^ instar larva in size. The sclerite DPa is absent on each of 1^st^ to 6^th^ abdominal segments of 1^st^ instar larva, but present on that of 3^rd^ instar larva. The sclerite DPp3 is absent on each of 2^nd^ to 7^th^ abdominal segments of 1^st^ instar larva, but present on that of 3^rd^ instar larva. The sclerite DPp4 is equipped with only one seta on each of 2^nd^ to 7^th^ abdominal segments of 1^st^ instar larva, but equipped with two setae on that of 3^rd^ instar larva. The sclerite LA is absent on each of 2^nd^ to 7^th^ abdominal segments of 1^st^ instar larva, but present on that of 3^rd^ instar larva. The sclerite LP is equipped with two setae on each of 1^st^ to 8^th^ abdominal segments of 1^st^ instar larva, but three on that of 3^rd^ instar larva. On the venter, the sclerite VA3 is absent on each of 1^st^ to 8^th^ abdominal segments of 1^st^ instar larva, but present on that of 3^rd^ instar larva. The sclerite VM2 on each of 2^nd^ to 5^th^ abdominal segments is absent in the first instar larva while present on that of the third instar larva. Additionally and likewise, the sclerite VM3 on each of 2^nd^ to 6^th^ abdominal segments is also absent in the first instar larva, while present on that of the third instar larva. The sclerite VP1 is absent on each of 1^st^ to 6^th^ abdominal segments of 1^st^ instar larva, but present on that of 3^rd^ instar larva. Similarly, the sclerite VP3 is absent on each of 1^st^ to 7^th^ abdominal segments of the 1^st^ instar larva, but present on that of 3^rd^ instar larva. The sclerite VP4 on each of 2^nd^ to 7^th^ abdominal segments of the 1^st^ instar larva is equipped with two setae, arranged in a longitude row, but only one seta on that of 3^rd^ instar larva.

## Supplementary Material

XML Treatment for Osmylus fulvicephalus

## Figures and Tables

**Figure 3a.**
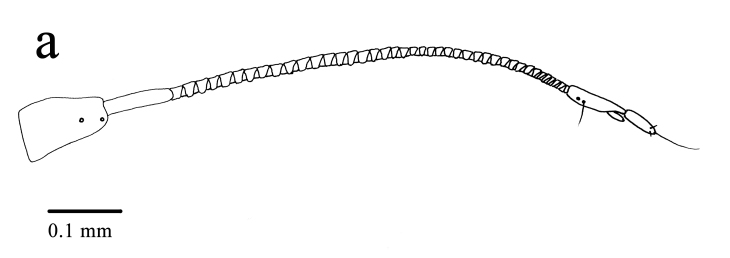
Antenna;

**Figure 3b.**
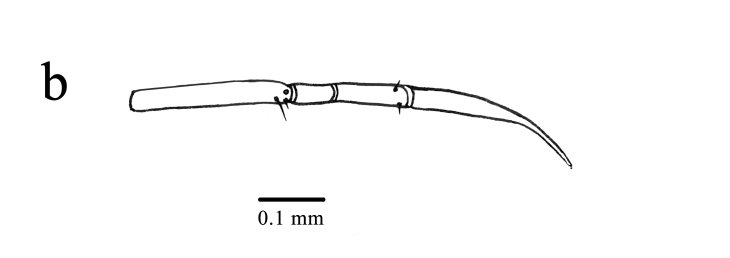
Labial palpi.
